# The Number of Regulatory B Cells is Increased in Mice with Collagen-induced Arthritis

**DOI:** 10.1515/biol-2019-0002

**Published:** 2019-03-20

**Authors:** Li Luo, Qing Liu, Shanshan Peng, Yan Meng, Wenjing Du, Demei Luo, Qian Wang, Jianbing Ding, Xunan Dong, Xiumin Ma

**Affiliations:** 1 State Key Laboratory Incubation Base of Xinjiang Major Diseases Research, The First Affiliated Hospital of Xinjiang Medical University, No. 137 South Liyushan Road, Urumqi 830011, Xinjiang Uygur Autonomous Region, Urumqi P.R. China; 2 The Fifth Affiliated Hospital of Xinjiang Medical University, No. 118 West Henan Road, Urumqi 830011, Xinjiang Uygur Autonomous Region, Urumqi P.R. China; 3 State Key Laboratory Incubation Base of Xinjiang Major Diseases Research, The First Affiliated Hospital of Xinjiang Medical University, Urumqi 830011, Urumqi P.R. China; 4 College of Basic Medicine, Xinjiang Medical University, Urumqi 830011, Urumqi P.R. China

**Keywords:** Bregs, IL-10, TGF-β, collagen-induced arthritis, immune regulation

## Abstract

The aim of this study is to investigate changes in regulatory B cells (Bregs) and the expression of related cytokines such as interleukin-10 (IL-10) and transforming growth factor (TGF)-β in a mouse model of collagen-induced arthritis (CIA). A total 20 DBA/1 mice (6-8 weeks old) were randomly divided into control and CIA disease groups. For the CIA disease group, animals were injected intradermally with chicken collagen type II and complete Freund's adjuvant. The calculated arthritis index score of the CIA group was significantly higher than that in control group. Hematoxylin and eosin staining showed tumid synovial cells with irregular arrangement and obvious hyperplasia, with a high degree of inflammatory cell infiltration in CIA model group. Cytometric bead array technology and quantitative RT-PCR indicated that the levels of IL-10 and TGF-β in serum, and synovial cells were significantly increased in the CIA group. The proportion of Bregs in the spleen of the CIA group was significantly increased compared to the control group. In conclusion, our findings demonstrate that the number of Bregs and the expression of TGF-β and IL-10 are enhanced in mice with CIA.

## 1 Introduction

Rheumatoid arthritis (RA) is a common autoimmune disease, which involves multiple organs but is most commonly manifest by joint inflammation [1]. RA tends to occur in women, and the incidence of RA peaks at 45 to 50 years old [2]. The disease is characterized by repeated episodes and high morbidity, and it is difficult to be cured [3]. Without proper treatment, joints in patients with RA can become deformed and even lose function [1, 2]. The pathogenesis of RA is complicated. B cells are among the most important cells in humoral regulation, and can influence the reaction of CD4 T cells, and function as antigen presenting cells, secreting an abundance of cytokines such as interleukin (IL)-4, IL-6, IL-10 and transforming growth factor (TGF)-β, etc. [4]. TNF-α induces apoptosis and necrosis [5]. In addition to the above listed cytokines, TNF-α is a significant contributor to the development of rheumatoid arthritis. Consequently, there are therapies that block TNF-α activity [6]. For a long time, researchers believed that B cells participated in immune regulation mainly by stimulating antibody secretion by downstream cells. However, special B cell subsets also have the function of regulating immune responses. B cells that exert a protective effect and inhibit immune responses are named regulatory B cells (Bregs). Bregs are a new kind of immune regulatory cell that have been discovered in recent years, and their role in autoimmune diseases has become intensively investigated [3, 7, 8, 9, 10]. It has been shown that Bregs can regulate or inhibit the effect of T cells, and that their dysfunction may cause autoimmune disease [11, 12]. Bregs promote immune tolerance and inhibit inflammation through a receptor mediated process [13]. Mizoguchi et al. report a subgroup of Bregs displaying a CD1d+CD5+CD19+ phenotype that produces IL–10, and is named B10. IL-10 is also understood to be important in maintaining Tregs in the body [14, 15]. Bregs are thought to maintain the balance of pro-inflammatory and antiinflammatory suppression, as well as regulate homeostasis via high expression of TGF-β and low expression of IL-6 [16, 17, 18]. Collagen-induced arthritis (CIA) produces a similar set of clinical symptoms and pathological features as RA, and the animal model of CIA is now the most commonly used model to study RA [19, 20, 21, 22]. In the present study, we explore the correlation between Bregs and RA immune inflammation, and examine the immune regulation and function mechanism of Bregs in RA.

## 2 Materials and methods

### 2.1 Animals

A total of 20 male DBA/1 mice (6-8 weeks old, 18-22 g weight) were bought from Beijing Weitong Lihua Experimental Animal Technical Co., Ltd. (Beijing, China), and housed in an environmentally controlled room with constant temperature and a 12-hour light/dark cycle. Mice were acclimated for fifteen days prior to experiment initiation. The mice were numbered, and randomly and evenly divided into experimental (CIA disease group) and control groups. Chicken CII (100 μg/mouse; Chondrex, Redmond, WA, USA) was emulsified in equal volumes of complete Freund’s adjuvant (Chondrex, Redmond, WA, USA) on ice to obtain 2 mg/ml solution. Each mouse in the CIA group was injected intradermally at the base of the tail with 100 μl of solution, while animals in control group received an equal volume of physiological saline. Immunization was repeated 21 days after the first injection. Arthritis development was monitored and scored in a blinded manner every second or third day.

The mice were sacrificed by cervical dislocation on day 42. Spleen and joint synovial tissues were snap frozen in liquid nitrogen and stored at -80°C. Joints were fixed in 4% paraformaldehyde for 24 hours, and transferred to decalcification buffer, and stored 4°C. Decalcification buffer was replaced every other day, for 7 to 10 days, until the bone was soft. The decalcified specimens were washed with phosphate-buffered saline, and dehydrated for paraffin-embedding. Then, 4-μm sections were prepared for histological examination.

**Ethical approval** The research related to animals use has been complied with all the relevant national regulations and institutional policies for the care and use of animals.

### 2.2 Evaluation of arthritis

According to the degree of joint swelling, mice in treatment groups were assessed every second or third day in a blinded manner. Standards were defined as follows: 0 points, normal joint without swelling or erythema; 1 point, slight swelling or redness; 2 points, moderate swelling at ankle or wrist joints; 3 points, severe erythema and swelling affecting the entire paw; 4 points, deformed paw or joint with ankylosis. The total scores for the four limbs ranged from 0 to 16 points.

### 2.3 Hematoxylin and eosin (HE) staining

Synovial tissues were collected from mice for HE staining. Synovial tissues were observed to determine whether inflammatory cell infiltration existed, whether synovial tissues were deformed, and whether articular cartilage was damaged.

### 2.4 Cytometric beads array (CBA) technology

The level of IL-10 expression in the serum of mice was tested by CBA technology. The results were analyzed by flow cytometry (BD Biosciences, Franklin Lakes, NJ, USA). Corresponding standard curve was plotted, and then the data were read according to the standard curve to calculate the level of IL-10.

### 2.5 Quantitative real-time polymerase chain reaction (qRT-PCR)

Before total RNA extraction, tissues (100 mg) were ground into powder using liquid nitrogen before addition of 1 ml Trizol (Thermo Fisher Scientific, Waltham, MA, USA) for lysis. After lysis, total RNA was extracted using the phenol chloroform method. The purity of RNA was determined by A260/A280 using ultraviolet spectrophotometry (Nanodrop ND1000, Thermo Scientific, Waltham, MA, USA). Then, cDNA was obtained by reverse transcription using PrimeScript RT Reagent Kit (Takara, Dalian, China) from 1 μg RNA and stored at -20°C. To measure TGF-β and IL-10 expression in tissues, SYBR Green qRT-PCR kit (Takara, Dalian, China) was employed. The full genetic sequences of TGF-β and IL-10 were acquired from GeneBank, and GAPDH was used as internal reference (Table 1).

**Table 1:** Primers and fragment lengths

Genes	Genbank accession No.	Primers	Fragment length (bp)
TGF-β	M13177.1	Forward: 5’GAGTTCACATGCGCCTTGAT3’	199
		Reverse: 5’TCGCTTTGTACAACAGCACC3’	
IL-10	NM_010548.2	Forward: 5’TGCTATGCTGCCTGCTCTTA3’	243
		Reverse: 5’TCATTTCCGATAAGGCTTGG3’	
GAPDH	AY618199.1	Forward: 5’AACTTTGGCATTGTGGAAGG3’	222
		Reverse: 5’CACATTGGGGGTAGGAACAC3’	

The PCR system (20 μl) included 10 μl 2 × SYBR Green PCR Premix, 0.5 μl upstream primer, 0.5 μl downstream primer, 2 μl cDNA template, and 7 μl ddH_2_O. PCR conditions were: initial denaturation at 95°C for 3 min; 40 cycles of denaturation at 95°C for 10 s, annealing at 55°C for 30 s and elongation at 72°C for 30 s. Fluorescence signals were determined to plot dissolution curve. Each sample was tested in triplicate.

### 2.6 Flow cytometry

Flow cytometry was used to determine the number of Bregs in the spleen of mice in CIA group. First, mononuclear cells were isolated from mouse spleen, and fluorescent tags of cell surface-specific antigen CD1d-PE mu (1 μl) antibody, CD5-PECy5 mu (1 μl) antibody, and CD19-FITC mu (1 μl) antibody were added. Then, 500 μl fixation / permeabilization solution was added. Flow cytometry (FACSVerse; BD Biosciences, Franklin Lakes, NJ, USA) was used to detect the number of Bregs after staining. Acquired flow cytometry data were analysed by software (BD Biosciences, Franklin Lakes, NJ, USA).

### 2.7 Statistical analysis

Results were analyzed using SPSS v17.0 software (IBM, Armonk, NY, USA). All data were expressed as means ± standard deviation. Data were tested for normality. Multigroup measurement data were analyzed using one-way ANOVA. In the case of homogeneity of variance, Least Significant Difference and Student-Newman-Keuls methods were used; in case of heterogeneity of variance, Tamhane’s T2 or Dunnett’s T3 method was used. Group t-test was used for comparative analysis between two groups, and differences were considered statistically significant if *P* < 0.05.

## 3 Results

### 3.1 Arthritis in the CIA group was more severe than that in the control group

To evaluate the severity of disease, arthritis index scores were calculated. Mice in the control group exhibited no joint swelling, while those in CIA group had paw redness and swelling, joint swelling, and restricted movement. In addition, the difference in arthritis scoring between the CIA and control groups was statistically significant (Table 2). This result suggests that arthritis in CIA group was more severe than that in control group.

**Table 2:** Arthritis index scores

Groups	Weight (g)	Swelling (paw)	Thickening (paw)	Limitation of activity	Scores
Control group	16.3 ± 2.2	0	0	0	0
CIA model group	15.8 ± 2.4	3.4 ± 0.6*	3.5 ± 0.5*	1.9 ± 0.9*	11.9 ± 1.9*

Note: *, P < 0.05 compared with control group.

### 3.2 Mice in the CIA group show increased inflammatory cells in synovial fluid of the joint

**Figure 1:**
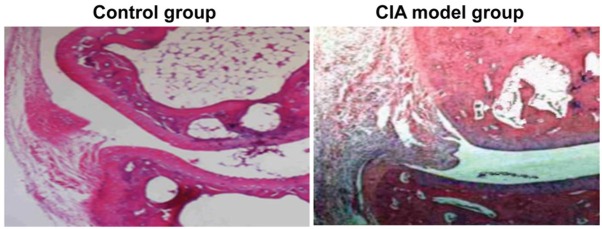
Inflammatory cells in synovial fluid of the joint. Hematoxylin and eosin staining was used to visualize the cells in control and CIA model groups. Magnification, ×400.

To observe synovial inflammatory cells, HE staining was performed. In the control group, synovial fluid contained 1-2 layers of synovial cells that were arranged neatly without hyperplasia, and no inflammatory cell infiltration was observed. In the CIA group, synovial cell edema, 3-5 layers of cells and obvious hyperplasia existed. Additionally, inflammatory cell infiltration and new blood vessels were observed (Figure 1). These results indicate that mice in CIA group have a large amount of inflammatory cells in synovial fluid of the joint.

### 3.3 Expression of IL-10 and TGF-β in diseased mice is enhanced

To compare the abundance of IL-10 and TGF-β in the serum between the CIA and control groups, CBA technology was used. The data showed that serum levels of IL-10 and TGF-β in the CIA group were significantly higher as compared to the control group (*P* < 0.05) (Table 3). This result suggests that the expression of IL-10 and TGF-β in the CIA group of mice is enhanced.

**Table 3:** Expression of IL-10 and TGF-β in serum

Groups	IL-10 (pg/ml)	TGF-β (pg/ml)
Control group	55.35 ± 6.34	22.96 ± 5.31
CIA model group	90.01 ± 9.61*	62.95 ± 11.21*

Note: *, P < 0.05 compare with control group.

### 3.4 High expression of IL-10 and TGF-β mRNA exists in the synovial membrane of mice with CIA

**Figure 2:**
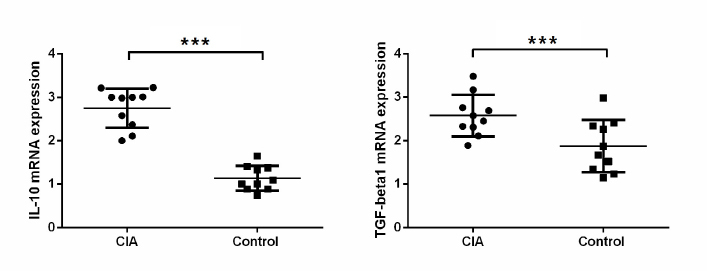
Relative expression of IL-10 and TGF-β mRNA in synovial membrane of CIA model mice. Quantitative real-time polymerase chain reaction was used to measure mRNA expression. ***, *P* < 0.05 compared with control.

To detect the expression of IL-10 and TGF-β mRNA, qRT-PCR was carried out. The mRNA levels of IL-10 and TGF-β in synovial tissue from mice in the control group were 0.99 ± 0.52 and 1.45 ± 0.53, respectively, while those in synovial tissue of mice in the CIA group were 2.43 ± 0.62 and 2.39 ± 0.64, respectively. Differences between the two groups were statistically significant (Figure 1Figure 2). These result suggest that high expression of IL-10 and TGF-β mRNA exists in the synovial membrane of mice with CIA.

### 3.5 The frequency of Bregs in the spleens of mice with CIA is elevated

**Figure 3:**
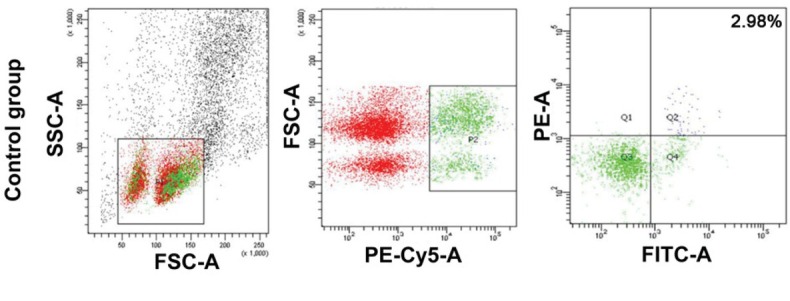
The percentage of Bregs in mouse spleen of control and CIA groups. Flow cytometry was employed to measure the number of Bregs in the spleens of mice in control and CIA model groups. Mononuclear cells were isolated from mouse spleen, and fluorescent tags of cell surface-specific antigen CD1d-PE mu (1 μl) antibody, CD5-PECy5 mu (1 μl) antibody, and CD19-FITC mu (1 μl) antibody were added. Then, 500 μl fixation / permeabilization solution was added.

To determine the number of Bregs in the spleen, flow cytometry was employed. As the phenotype of Breg cells in mice was CD1d^high^CD5 ^+^ CD19^high^, the proportion of Bregs in the spleen from the control group was 2.98 ± 0.93%, while that in CIA model group was 8.88 ± 2.17%. The difference was statistically significant (*P* < 0.05) (Figure 3). This result indicates that the number of Bregs in the spleens of mice with CIA is elevated.

## 4 Discussion and Conclusions

RA is a complicated autoimmune disease with relatively high morbidity, causing a heavy burden to families and the society. RA is characterized by elevated expression of auto-antibodies such as rheumatoid factor and ring citrulline peptide antibodies [23]. Numerous studies have demonstrated that both cellular immunity and humoral immunity play important roles in the development of RA [24, 25]. It has been shown that the role of T cells and their cytokines play vital roles in RA pathogenesis [26, 27]. It is believed that B cells are involved in the immune response through production of antibodies and a variety of cytokines such as IFN-gamma, TNF-alpha, IL-4, and IL-13. In addition, B cells also play an important role in humoral immune system. Bregs are a newly discovered type of cell [28, 29], and are thought to influence the disease immune response and inflammatory reactions [30, 31, 32] by playing a suppressive role in the body’s immune response. Bregs promote immunosuppression mainly through the secretion of IL-10 and TGF-β [33, 34] by inhibiting or reducing the activation of T cells, and enhancing the production of related cytokines. This suggests that Bregs can reduce pathogen elimination capacity and increase immunologic tolerance. Bregs alleviate severe autoimmune diseases, including contact hypersensitivity, experimental autoimmune encephalomyelitis (EAE), chronic enteritis and collagen-induced arthritis [35,36]. The influence of Bregs on the secretion of IL-10 is found in models of contact dermatitis and EAE Bregs. A subset of B cells, known as B10, arise in the spleen and other lymphoid tissues, and are marked as CD1dhiCD5+CD19hi. The development of Bregs influences disease through secreting IL-10 to regulate T cell differentiation. Mice with defective Bregs secret less IL-10, which leads to elevated Th1 responses [37, 38].

By detecting Bregs and specific cytokines in a mouse model of CIA, the present study shows the dynamic trends of Bregs in the process of RA pathogenesis. The CIA group showed a large number of infiltrated inflammatory cells found in affected joints, as well as new blood vessel formation. Concordantly, the mRNA expression of TGF-β and IL-10 in the synovial membrane of CIA mice was significantly increased. In addition, the frequency of Bregs in splenic lymphocytes was increased in CIA mice. The presence of TGF-β and IL-10 in the serum was also markedly elevated in CIA mice. IL-10 secreted by Bregs is known to reduce cell surface expression of class II major histocompatibility complex molecules [39, 40, 41], and the expression of cytokines such as TNF-alpha [42], reduce effector function in T cells and/or regulatory T cells [43], regulate Th1/Th2 balance that inhibits positive immune response, and dampen inflammatory responses in CIA synovial injury. Meanwhile, excessive expression of IL-10 can also restrain the secretion of IL-12 from macrophages and dendritic cells. Consequently, the proliferation and differentiation of T cells and positive immune responses are inhibited. The present study has also discovered that IL-10 levels in CIA mice is increased significantly, being consistent with the report by Mizoguchi [44]. T cells can be induced to CD4+CD25+Foxp3+ regulatory T cells (iTreg) by TGF-β that is produced by Bregs. In addition, iTreg maintains stability in inflammatory conditions, inhibiting effector T cells through by expression of Foxp3, and also suppresses Th2 and Th17 cells, thereby inhibiting the destructive immune response in joints of CIA mice. In addition, iTreg signaling pathways inhibit osteoclasts by contacting NF-kB cells in a dose-dependent manner, and reduce bone erosion and damage caused by osteoclasts [45]. The present study shows that TGF-β in a CIA mouse model is increased through the induction of iTreg cells which regulate negative immune responses, finally leading to the occurrence of RA. In conclusion, Bregs and the expression of related factors are important in the pathogenesis of CIA. After being activated, Bregs can produce a large amount of TGF-β and IL-10, which can lower specific immune response levels and limit inflammatory reactions.
